# Effect of concurrent resistance-aerobic training on inflammatory factors and growth hormones in children with type 1 diabetes: a randomized controlled clinical trial

**DOI:** 10.1186/s13063-023-07553-0

**Published:** 2023-08-12

**Authors:** Marzieh Nazari, Ramin Shabani, Afagh Hassanzadeh-Rad, Mohammad Ali Esfandiari, Setila Dalili

**Affiliations:** 1 Faculty of Humanities, Rasht Branch, Islamic Azad University, Rasht, Iran; 2 Department of Exercise Physiology, Faculty of Humanities, Rasht Branch, Islamic Azad University, Rasht, Iran; 3 Pediatric Diseases Research Center, Guilan University of Medical Sciences, Rasht, Iran; 4 School of Medicine, Guilan University of Medical Sciences, Rasht, Iran; 5 Virtual School of Medical Education and Management, Shahid Beheshti University of Medical Sciences, Tehran, Iran

**Keywords:** Exercise, Child, Diabetes mellitus, Interleukins, Insulin-like growth factor I, Glycated hemoglobin, Randomized controlled trial

## Abstract

**Background:**

Exercise training is a major factor in controlling type 1 diabetes mellitus (T1DM) in children. The present study aimed to assess the effect of concurrent resistance-aerobic training on selected inflammatory factors and hormones related to blood glucose homeostasis in children with T1DM.

**Methods:**

In this randomized controlled clinical trial, 40 children (with the mean age of 11.11 ± 2.29 years) were randomly assigned to an experimental (*N* = 20) or control group (*N* = 20). They underwent a 16-week training program, composed of concurrent resistance-aerobic training performed intermittently for 60 min three times a week. Before and after training, blood samples were analyzed for glucose homeostasis, selected inflammatory factors, and growth factors. Data were analyzed by paired *t*-test and analysis of covariance (ANCOVA) in IBM SPSS version 22.

**Results:**

The exercise training intervention reduced fasting blood sugar index (*P* = 0.002) and glycosylated hemoglobin significantly (*P* = 0.003). The growth hormone levels were increased significantly only in the experimental group (*P* = 0.037), whereas no significant difference was noted in the insulin-like growth factor-1 (*P* = 0.712). It was also found that interleukin-1β and high-sensitivity C-reactive protein did not change in the experimental or control group as compared to the pretest (*P* > 0.05).

**Conclusion:**

As it was shown, it seems that concurrent resistance-aerobic training may improve blood glucose homeostasis and growth hormone. Therefore, these findings may suggest the benefit from exercise training of moderate intensity in children with T1DM. Besides, we recommend undertaking further clinical trials to determine if the exercise training was effective.

**Trial registration:**

This study was registered in the Iranian Registry of Clinical Trials under the code IRCT20150531022498N30: https://en.irct.ir/trial/41031. Registered on July 26, 2019. All experiments on the participants were following the Declaration of Helsinki.

**Supplementary Information:**

The online version contains supplementary material available at 10.1186/s13063-023-07553-0.

## Background

Type 1 diabetes mellitus (T1DM) is a chronic inflammatory autoimmune disease caused by the destruction of pancreas beta cells and causing life-long dependence upon external insulin [1]. Generally, it results from a multi-stage process initiated from the very early stages of life in children [2]. A recent report estimated that over 1.1 million children and teenagers are suffering from T1DM worldwide [3]. In addition to the daily injection of external insulin, the control of blood sugar, nutrition, and physical activity are major issues in controlling this disease. Most people with T1DM take a more sedentary lifestyle than healthy individuals due to the fear of hypoglycemia and blood glucose control. Exercise-induced hypoglycemia and hyperglycemia are commonplace among these people [4].

Considering the relationship between blood glucose and inflammation, blood sugar plays a critical role in inducing and aggravating inflammatory processes and there is a complicated relationship among cytokines, inflammation, and adaptive responses in maintaining blood homeostasis [5]. Indeed, diabetes is characterized by intra-islet expression of inflammatory mediators, especially cytokine interleukin-1 (IL-1), which causes cell apoptosis, progressive cell loss, and diabetes. IL-1 plays a key role as a mediator of autoimmune diseases [6].

A recent study demonstrated the role of physical activity in mitigating inflammatory responses. There are reports that regular exercise has anti-inflammatory effects and suppresses low-degree systemic inflammation [7–10]. However, some researchers have observed an increase in pro-inflammatory cytokines during high-intensity exercises [10, 11]. On the other hand, the lack of insulin and inflammation impair the performance of insulin-like growth factor 1 (IGF-1) and affects the GH-1GF axis, and it has been shown that physical activity raises growth hormone and IGF-1 [12].

It has been shown that resistance training can strengthen children and accelerate their maturity [13]. In contrast, aerobic training influences the activation of muscles and can improve insulin action in each muscular fiber. In this respect, even though Pilates is a resistance training, it has no side-effect for children and juveniles and some previous studies revealed the positive impacts of resistance training on children and patients with T1DM [14–16]. However, there is no consensus on the effectiveness of these training activities on patients with T1DM [11, 17, 18]. Since the potential advantages of this training type to children and juveniles have not been assessed adequately [15] and modifying immune responses to sport in children with T1DM are mostly unknown, the present study aimed to shed light on the effect of concurrent resistance and aerobic training on selected inflammatory factors and hormones related to blood glucose homeostasis in children suffering from T1DM.

## Methods

### Design and settings

This randomized paralleled controlled trial was conducted between February 2019 and June 2019 on children with T1DM aged 8–14 years who visited 17-Shahrivar Children’s Hospital, Iran. The study was approved by the Ethics Committee of Islamic Azad University of Rasht, Iran, under the ethics approval code of IR.IAU.RASHT.REC.1398.011. It was also registered in the Iranian Registry of Clinical Trials under the code of IRCT20150531022498N30: https://en.irct.ir/trial/41031. The written informed consent letter was obtained from the parents/guardians before enrollment. All experiments on the participants followed the Declaration of Helsinki.

Participants were recruited to the study if they met the following inclusion criteria: (1) pediatric T1DM, (2) glycosylated hemoglobin (Hb A1c) ≥ 7, (3) age 8–14 years, and (4) duration of diabetes for at least 1 year.

The exclusion criteria were severe hypoglycemia (< 50 mg/Dl), unconsciousness, and cardiac autonomic neuropathy. The participants who were absent for more than 5 sessions were excluded from the research as well. All the participants included in the randomization process (20 in the exercise group and 20 in the control group) completed the study and no participants dropped out.

The randomization process was done based on the red and blue balls inside the box that were randomly taken by the participants (the Red Orb and the Blue Orb of the experimental and control group). This process was supervised by an independent researcher. The control group had no exercise. The participants had to follow the dietary recommendations throughout the study. Dietary intakes were assessed using a 3-day food record (two weekdays and one weekend) at the baseline and the end of the study in both groups. Dietary recommendations were made with an emphasis on the use of a variety of foods (e.g., whole grains, fat-free, fruits, vegetables, poultry, fish, and nuts).

To find out the tolerance of the exercise method by the subjects and to define sample size, the training protocol was first applied in a pilot study on five male and female patients with T1DM for 2 weeks. These patients were not included in the final sampling. In the end, they showed mild symptoms, like sourness, because the training was initiated at a low intensity. Therefore, no intolerable side effect was noted. The results revealed that the training protocol can be executed on diabetic patients aged 8–14 years.

### Sample size calculation

Regarding the shortage of evidence on the effect of training on children with T1DM, the sample size (*n* = 20 in each group) was determined based on our pilot study using the G-Power software (ver. 3.1.9.2) with an error level of *α* = 0.05, error *β* = 80.0, and the effect size of 0.8.

### Measurements

We took measurements one week before the exercise and 48 h after the intervention. Body composition consisting of height, weight, body mass index, and fat percentage was measured by an Inbody 3.1 (South Korea).

Regarding the effects of hormones at puberty, we used the tanner staging as a factor of homogenization. The self-reported five-step tanner stage image including breast and genital development for girls and genital development for boys was used to determine the stage of puberty [19]. The subjects were in stages 1 to 5 of puberty based on the Tanner staging.

Growth hormone, IGF1, IL-B, and hs-CRP were the main outcomes assessed in this study. For the blood test of both experimental and control groups, a 5 ml sample was collected from the arm vein in a seated position at rest. Fasting blood glucose (FBS) and Hb A1c were measured with kits from the Pars Azmun Company (Iran, with a sensitivity of 1 mg dL^−1^) and Biosystem (Spain, with a sensitivity of 1 mg dL^−1^) by biochemical method, respectively. IGF-1 and growth hormone were measured by the commercial kit of Dimatera (Italy) with the sensitivity of 1 and 0.1 ng mL^−1^ by the ELISA method, respectively. The test kit of Diaclone (France) was used to measure IL-1β by the ELISA method. Finally, hs-CRP was measured by a test kit made by the Pars Azmun Company (Iran) by the biochemical method.

### Design of exercise program

The training program was 16 weeks of interval concurrent resistance-aerobic exercises for 60 min three times a week. Each session began with 10 min of warming and ended with 5 min of cooling. The subjects first performed resistance training. Then, the aerobic exercises were performed, including V-forward, V-back, standing lateral, standing crunch, and march at 50–75% of the maximum heart rate. The resistance training program included Pilates composed of stretching hand to four sides, single leg stretch, breast stretch, cat stretch, and sit-up performed in 2–3 sets of 8–12 repetitions with a 30-s break between the sets. Furthermore, the body weight-bearing training was composed of lunges and squats with body weight, one-legged kickbacks and standing calf raise with body weight, and push-up with the same sets and repetitions as Pilates [11, 20, 21] (Table 1).

**Table 1:** Concurrent training protocol

**Exercise**	**Week**	**Rest**	**Sets/repetitions**	**Duration**	**Intensity**
Pilates (5 exercises)	1–8	30 s	1–2 s 8–10 r	10 min	------
	9–16	30 s	2–3 s 10–12 r	20 min	------
Body weight bearing (5 exercises)	1–8	30 s	1–2 s 8–10 r	10 min	------
	9–16	30 s	2–3 s 10–12 r	20 min	------
Aerobic training (5 exercises)	1–8	1 min between sets	2 set (5 min + 5 min)	10 min	50–60% HR
	9–16	2 min between sets	2 set (10 min + 10 min)	20 min	65–75% HR

*min* minutes, *sec* second, *HR* heart rate

### Statistical analysis

Data were analyzed by IBM SPSS Statistics for Windows, Version 22.0. Armonk, NY: IBM Corp. The normal distribution of the data was checked by the Kolmogorov–Smirnov test, and regarding the normally distributed data, they were assessed by paired *t*-test and the analysis of covariance (ANCOVA). A *P*-value < 0.05 indicated statistical significance.

## Results

**Fig. 1:**
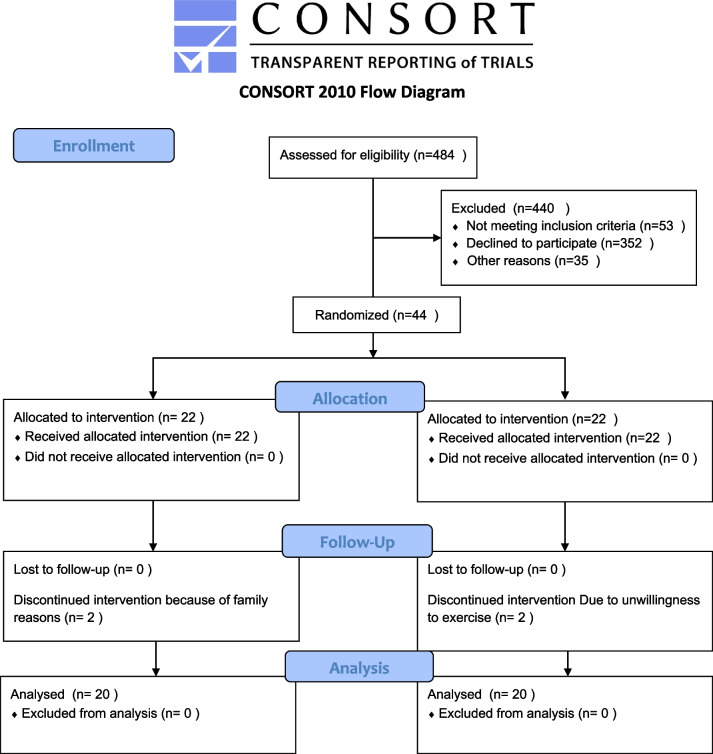
Consort flow diagram

Regarding the eligibility criteria, 44 participants were randomly assigned to an experimental (*n* = 22) and a control group (*n* = 22) to participate in a 16-week trial. During the trial, four children were excluded for family reasons. The remaining were categorized into an experimental (20 subjects) and a control group (20 subjects) (Fig. 1, Additional file 3). Table 2 presents the demographic characteristics of the participants.

**Table 2:** Descriptive data of the subjects

**Variable**	**Exercise** **(*n* = 20)** **Mean ± SD**	**Control** **(*n* = 20)** **Mean ± SD**
Age (years)	11.22 ± 1.90	11.00 ± 2.67
Boys/girls (*n*)	8/12	11/9
Diabetes duration (years)	3.04 ± 1.83	3.07 ± 1.87
Height (cm)	145.15 ± 11.75	141.22 ± 19.27
Tanner stage	2.45 ± 0.99	2.35 ± 1.13
Hb A1c (%)	7.98 ± 1.02	7.26 ± 1.51
BMI (Kg/m^2^)	18.96 ± 4.11	17.28 ± 1.87

*BMI* body mass index

The changes in blood glucose, selected inflammatory factors, and hormone indices in groups during the pretest and posttest are provided in Table 3. Based on the results, the experimental group exhibited a significant decline in blood glucose index (*P* < 0.012), Hb A1c (*P* < 0.001), and growth hormone (*P* < 0.037) after 16 weeks of concurrent training. Also, the results of the ANCOVA test showed that the experimental and control groups differed in blood sugar index and Hb A1c significantly (*P* = 0.002 and *P* = 0.003), whereas no significant difference was observed in growth hormone between the groups. In addition, IGF-1 did not show any significant changes in either group despite its increase (*P* > 0.05).

**Table 3:** Changes in blood indices of the study subjects, from baseline to the end of the study

**Variable**	**Exercise (*n* = 20)**				**Control (*n* = 20)**				
	**Before**^**a**^	**After**^**a**^	**MD(95% CI)**	***P***^**b**^	**Before**^**a**^	**After**^**a**^	**MD(95% CI)**	***P***^**b**^	***P***^**c**^
**Glucose homeostasis**									
FBS (mg/dl)	163.10 ± 78.9	126.05 ± 66.69	37.05(8.99 to 65.10)	0.012*	165.25 ± 52.99	168.60 ± 54.47	03.35 (− 10.67to 3.97)	0.351	0.002*
Hb A1c (%)	7.98 ± 1.02	7.83 ± 1.00	0.712(0.180 to 1.243)	< 0.0001*	7.26 ± 1.51	8.15 ± 1.12	− 0.31 (− 0.72 to 0.086)	0.117	0.003*
**Inflammatory factors**									
IL-1β	4.921 ± 7.418	6.175 ± 7.074	− 1.25 (− 2.72 to 0.215)	0.9	3.687 ± 9.936	3.396 ± 5.664	0.29 (− 5.27 to 5.85)	0.914	0.209
hs-CRP	0.086 ± 020	0.151.0 ± 0.344	− 0.065 (− 0.225to 0.095)	0.406	0.187 ± 0.426	0.188 ± 0.428	− 0.001 (− 0.006to 0.003)	0.562	0.418
**Hormonal indicators**									
IGF-1 (ng/ml)	222.46 ± 139.99	227.02 ± 109.77	− 4.55 (− 40.17 to 31.06)	0.712	203.96 ± 79.77	205.79 ± 79.77	− 1.82 (− 9.16 to 5.51)	0.609	0.608
GH (ng/ml)	1.39 ± 2.33	2.30 ± 2.97	− 0.90 (− 1.75 to − 0.59)	0.037*	0.96 ± 0.89	1.79 ± 2.01	− 0.82 (− 1.68 to − 0.031)	0.058	0.896

*Hb A1c* hemoglobin A1c, *FBS* fasting blood sugar, *IL* interleukin, *IGF-1* insulin-like growth factor-1, *GH* grows hormones, *MD* mean difference, *CI* confidence interval

^a^All values are mean ± SD

^b^*P* value for paired *t* test

^c^*P* value for ANCOVA; adjusted for baseline values

*Statistically significant

In terms of IL-1β and hs-CRP, it was found that none were changed in the experimental and control groups significantly when compared to the pretest (*P* > 0.05). The between-group difference in the posttest IL-1β and hs-CRP was not significant either (*P* = 0.209 and *P* = 0.418, respectively) (Table 3).

## Discussion

The present study assessed the effect of concurrent resistance-aerobic training on selected inflammatory factors and hormones related to blood glucose homeostasis in children with T1DM. Based on the results, the experimental group had a significant decline in blood glucose index and Hb A1c after 16 weeks of concurrent training. The growth hormone index was enhanced significantly only in the experimental group. On the other hand, although the training protocol increased IGF-1, the increase was not statistically significant. The results, however, implied that despite the decreased blood sugar indices and enhanced growth hormone in the intervention group, it did not affect inflammatory markers in these patients. To the best of our knowledge, this is the first study reporting the effect of concurrent resistance and aerobic training on inflammatory indices in children with T1DM. Since it is crucial to control T1DM in children and hinder its complications, the findings should be considered in pediatric diabetes control programs. This concurrent training is recommended for children as it is both low-cost and highly useful in preventing the consequences of the disease in the future.

We found that Hb A1c was statistically significant, but it changed very slightly. This may occur because our participants already have had a good apparent control on their blood glucose so that they had relatively low HbA1c at the beginning. Regarding fast blood glucose, a significant decline was observed after the training. This can be related to moderate intensity and long duration of the exercise training. On the other hand, neither blood glucose index nor Hb A1c changed in the control group. It can be concluded that exercise training provokes various responses in blood glucose concentration depending on its intensity and duration, so it was shown that low-intensity sprint exercises and training with weight increased blood glucose. Similar to the results of the current study in terms of blood sugar and Hb A1c, Fountaine et al. reported that moderate-intensity continuous training and high-intensity intermittent sprint training contributed to maintaining target blood sugar levels in adults with T1DM [22]. Likewise, Lascar et al. reported a decrease in Hb A1c, improvement in insulin sensitivity, and enhancement of the performance of beta-cells after 12 months of physical activity at a moderate to severe intensity (150–240 min) in newly diagnosed adult type 1 diabetes patients aged 16–60 years [23]. Also, a study mentioned that 3 weeks of aerobic training + resistance training reduced the blood glucose. Considering the reduction of blood sugar and Hb A1c, it can be concluded that exercise and contraction have a semi-insulin function and transport glucose into cells to be spent on energy generation. The mechanism may be associated with membrane permeability to glucose by glucose transporter proteins (GLUT4) and the increased gene expression or the activity of various proteins involved in the cascade of insulin signaling, the increase in capillary density, and the activity of glycogen synthase in muscular contraction [23]. Therefore, when the training is increased, GLUT4 increases, and it improves the translocation of glucose into the cells by the pathways that are not dependent on insulin.

However, Landt et al. did not show any significant changes in the blood glucose level of children with T1DM aged 11 after 5 months of aerobic training [24], which is inconsistent with our findings. They attributed the lack of any variations in the glycosylated index to performing just training and neglecting the food diet and insulin level. The reasons for the inconsistency of their findings with ours can be sought in the duration of training (12 weeks in their research vs. 16 weeks in ours), training protocol (aerobic and resistant training separately vs. simultaneous training), and training intensity.

On the other hand, the present study revealed that the growth hormone levels were significantly increased in both groups. Nevertheless, this training protocol failed to increase IGF-1 significantly. In contrast, Færch et al. reported a negative relationship between IGF-1 and glycemic control in patients with T1DM [25]. It has been shown that the serum IGF-1 level in newly diagnosed children with T1DM was reduced and treatment with insulin increased the IGF-1 level in these patients. Also, another research on the effect of high-intensity continuous and intermittent exercise on IGF-1 in children with T1DM reported that the exercise had an increasing impact on IGF-1 in these patients [26]. The authors concluded that the effectiveness of the exercise depended on the fitness and exercise parameters of the subjects.

Physical activities and appropriate exercise training can affect final height growth, muscle mass increase, and body fat decrease via inducing the secretion of growth hormone and, subsequently, IGF-1. On the other hand, the growth of various body systems in children and juveniles closely depends on the level of these hormones. Concerning the increase in these hormones in the present study, it can be noted that adaptability to training boosts growth hormone and IGF-1 transporters in the body, so their increase after 16 weeks of moderate-intensity training can be attributed to this adaptability.

Proteins bound to growth hormone and IGF-1 are influential in their performance [27]. Although the subjects here were children with T1DM and, due to their specific condition, the intervention was developed at a moderate intensity, the training variables including intensity and duration could well induce the response of anabolic hormones such as growth hormone and cause adaptation to them.

On the other hand, the study revealed that inflammatory indices were increased in both groups. This finding supported the results of a study on the effect of high-intensity aerobic and resistance training in adults with T1DM [28]. This result may be ascribed to the increase in blood glucose during physical activity in which case this stress itself increases and activates pro-inflammatory cytokines. This stress can, however, be offset by the activation of an anti-inflammatory response and daily moderate-intensity exercise training along with an optimal diet.

A recent study by Boff et al. on the effect of high-intensity interval training (HIIT) and moderate-intensity continuous training on adults with T1DM indicated that hs-CRP was increased in both protocols. The training protocols were performed in stationary cycle ergometers at 50–85% high intensity and 50% moderate intensity for 40 min for 8 weeks at a frequency of three times a week [10]. In this respect, it can be said that activity in stress and HIIT can aggravate inflammatory factors [26]. In both studies, training was in the form of HIIT. In contrast, another study reported a decline in cytokines during exercise training. Zebrowska et al. explored the effect of continuous training and HIIT on inflammatory responses in adults with T1DM and revealed that the training interventions reduced IL-1β and TNF-α in these patients [26]. To account for the finding, they stated that intermittent training reduces inflammation by stabilizing and making a balance in the secretion of growth factors and this improves vascular performance in T1DM. The disagreement between our findings and Zebrowska et al. may be related to the training protocol (high-intensity continuous training versus moderate-intensity concurrent training) and subject type (adults with T1DM vs. children with T1D). To describe its mechanism, it can be said that exercise increases growth hormone, thereby increasing cell-beta proteins and protecting beta-cells against apoptosis caused by pro-inflammatory factors, especially IL-1β. Nevertheless, these changes are dependent on training intensity, type, and duration. It seems that the lack of any changes in this index in the present study is related to the low intensity and duration of the training or the low sample size.

This study had some strengths and limitations. We indicated the tanner staging and participants in the groups were evenly distributed by tanner stages. We also assessed the effect of Pilates training, which is low-cost, healthy, and safe, especially for children. They also need no specific instrument and are readily available everywhere. However, there were some limitations. Future research needs to use random sampling with larger sample sizes. Nonetheless, the participants of both groups were selected homogenously. The participants enrolled in the study already had very good glucose control. It seems that longer exercise training should be performed to improve effectiveness.

## Conclusions

A 16-week moderate-intensity concurrent resistance-aerobic training program may contribute to improving blood glucose homeostasis in children with T1DM. However, there was no effect on hormone indices and selected inflammatory factors. Thus, given the significance of controlling T1DM in children and preventing its consequences in the future, the findings of the study should be considered when developing pediatric diabetes control programs. The concurrent training method is recommended to prevent the implications of the disease in children’s future since it is low-cost and beneficial. Besides, we recommend undertaking definitive clinical trials sufficiently powered to determine if the exercise training was effective.

### Supplementary Information

**Additional file 1.** Persian protocol summary: Persian protocol.

**Additional file 2.** English protocol summary: English protocol. Ethics approval in Persian: Ethics approval in Persian. Ethics approval in English: Ethics approval in English.

**Additional file 3.** Consort checklist: CONSORT 2010 Checklist.

## Data Availability

All data generated or analyzed during this study are included in this published article and supplementary information files.
