# Supplementary material for: Effect of concurrent resistance-aerobic training on inflammatory factors and growth hormones in children with type 1 diabetes: a randomized controlled clinical trial
Source: Trials. 2023 Aug 12;24:519. doi: 10.1186/s13063-023-07553-0 (PMC10422817; doi:10.1186/s13063-023-07553-0)
Supplement: Supplementary file 1 — Additional file 1. Persian protocol summary: Persian protocol. [file 13063_2023_7553_MOESM1_ESM.docx]

**اثر تمرینات ترکیبی بر فاکتورهای التهابی و سطوح هورمون های مرتبط با هموستاز گلوکز خون در کودکان مبتلا به دیابت نوع 1**

**چکیده پروتکل**

هدف ازمطالعه

اثر تمرینات ترکیبی بر فاکتورهای التهابی و سطوح هورمون های مرتبط با هموستاز گلوکز خون در کودکان مبتلا به دیابت نوع 1

طراحی

در این مطالعه، 44 کودک مبتلا به دیابت نوع 1 به صورت داوطلبانه حضور خواهند داشت که به صورت تصادفی به دو گروه 22 نفره تجربی و 22 نفره شاهد تقسیم می شوند.

نحوه و محل انجام مطالعه

پژوهش حاضر یک مطالعه نیمه تجربی بر روی کودکان مبتلا به دیابت نوع 1 شهر رشت است. آزمودنی ها به مدت 16هفته در برنامه تمرینات ترکیبی مقاومتی- هوازی شرکت خواهند کرد. خونگیری پس از 12 ساعت ناشتایی و قبل و پس از 16 هفته تمرین ترکیبی صورت خواهد گرفت.

شرکت کنندگان/شرایط ورود و عدم ورود

معیارهای ورود: داشتن دامنه سنی 15-8 سال، A1c بالای 7، کودکان با دیابت نوع 1، عدم فعالیت ورزشی منظم شرایط عدم ورود: بیش از سه جلسه غیبت از تمرینات

گروه‌های مداخله

گروه مداخله: گروه تمرینات ترکیبی: شانزده هفته ، سه جلسه در هفته. تمرینات هوازی با شدت 50 تا 75 درصد حداکثر ضربان قلب، بیست دقیقه، تمرینات پیلاتس سه ست با 6 تا 12 تکرار و تمرینات با تحمل وزن بدن به مدت بیست دقیقه انجام شد. گروه شاهد: بدون مداخله

متغیرهای پیامد اصلی

اینترلوکین 1 بتا، پروتئین واکنشگر C ، فاکتور رشد شبه انسولینی، هورمون رشد، کورتیزول، هموگلوبین گلیکوزیله، قند خون ناشتا، شمارش کامل خون

**اطلاعات عمومی**

علت بروز رسانی

نام اختصاری

اطلاعات ثبت در مرکز

شماره ثبت کارآزمایی در مرکز: **IRCT20150531022498N30**

تاریخ تایید ثبت در مرکز: **2019-07-26, ۱۳۹۸/۰۵/۰۴**

زمان‌بندی ثبت: **retrospective**

آخرین بروز رسانی: **2019-07-26, ۱۳۹۸/۰۵/۰۴**

تعداد بروز رسانی‌ها: **0**

تاریخ تایید ثبت در مرکز

2019-07-26, ۱۳۹۸/۰۵/۰۴

اطلاعات تماس ثبت کننده

**نام**

رامین شعبانی

**نام سازمان / نهاد**

دانشگاه آزاد اسلامی

**کشور**

جمهوری اسلامی ایران

**تلفن**

+98 13 3375 2715

**آدرس ایمیل**

shabani@iaurasht.ac.ir

وضعیت بیمار گیری

**بیمار گیری تمام شده**

منبع مالی

تاریخ شروع بیمار گیری مورد انتظار

2019-02-09, ۱۳۹۷/۱۱/۲۰

تاریخ پایان بیمار گیری مورد انتظار

2019-07-22, ۱۳۹۸/۰۴/۳۱

تاریخ شروع بیمارگیری تحقق یافته

خالی

تاریخ پایان بیمارگیری تحقق یافته

خالی

تاریخ خاتمه کارآزمایی

خالی

عنوان علمی کارآزمایی

اثر تمرینات ترکیبی بر فاکتورهای التهابی و سطوح هورمون های مرتبط با هموستاز گلوکز خون در کودکان مبتلا به دیابت نوع 1

عنوان عمومی کارآزمایی

اثر تمرینات ترکیبی در کودکان مبتلا به دیابت نوع 1

هدف اصلی مطالعه

پیشگیری

شرایط عمده ورود و عدم ورود به مطالعه

**شرایط عمده ورود به مطالعه قبل از تصادفی سازی:**

داشتن دامنه سنی 15-8 سال A1c بالای 7 کودکان با دیابت نوع 1 عدم فعالیت ورزشی منظم

**شرایط عمده عدم ورود به مطالعه قبل از تصادفی سازی:**

بیش از سه جلسه غیبت از تمرینات

سن

از سن **8 ساله** تا سن **15 ساله**

جنسیت

هر دو

فاز مطالعه

مصداق ندارد

گروه‌های کور شده در مطالعه

*اطلاعات موجود نیست*

حجم نمونه کل

حجم نمونه پیش‌بینی شده: **35**

تصادفی سازی (نظر محقق)

اختصاص تصادفی به گروه‌های مداخله و کنترل

توصیف نحوه تصادفی سازی

آزمودنی ها در 2 گروه (یک گروه کنترل و یک گروه تجربی) به روش تصادفی ساده تقسیم می شوند. این نمونه گیری براساس گوی های قرمز و آبی داخل جعبه که توسط آزمودنی ها به طور تصادفی برداشته می شود (گوی قرمزگروه شاهد و گوی آبی گروه تجربی) انجام خواهد شد.

کور سازی (به نظر محقق)

کور نشده است

توصیف نحوه کور سازی

دارو نما

ندارد

اختصاص به گروه‌های مطالعه

موازی

سایر مشخصات طراحی مطالعه

**کد ثبت در سایر مراکز ثبت بین‌المللی**

خالی

**تاییدیه کمیته‌های اخلاق**

**1**

کمیته اخلاق

**نام کمیته اخلاق**

کمیته اخلاق دانشگاه آزاد اسلامی واحد رشت

**آدرس خیابان**

رشت، پل تالشان، دانشگاه آزاد اسلامی واحد رشت

**شهر**

رشت

**استان**

گیلان

**کد پستی**

۴۱۴۷۶۵۴۹۱۹

تاریخ تایید

2019-07-19, ۱۳۹۸/۰۴/۲۸

کد کمیته اخلاق

IR.IAU.RASHT.REC.1398.011

**2**

کمیته اخلاق

**نام کمیته اخلاق**

کمیته اخلاق دانشگاه آزاد اسلامی واحد رشت

**آدرس خیابان**

رشت، پل تالشان، دانشگاه آزاد اسلامی واحد رشت

**شهر**

رشت

**استان**

گیلان

**کد پستی**

۴۱۴۷۶۵۴۹۱۹

تاریخ تایید

2017-09-19, ۱۳۹۶/۰۶/۲۸

کد کمیته اخلاق

IR.IAU.RASHT.REC.1396.168

**بیماری‌های (موضوعات) مورد مطالعه**

**1**

شرح

دیابت نوع 1

کد ICD-10

E10

توصیف کد ICD-10

Type 1 diabetes mellitus

**متغیر پیامد اولیه**

**1**

شرح متغیر پیامد

اینترلوکین 1 بتا

مقاطع زمانی اندازه‌گیری

قبل و بعد از اتمام مداخلات

نحوه اندازه‌گیری متغیر

با استفاده از کیت الایزا

**2**

شرح متغیر پیامد

پروتئین واکنش گر c

مقاطع زمانی اندازه‌گیری

قبل و بعد از اتمام مداخلات

نحوه اندازه‌گیری متغیر

با استفاده از کیت الایزا

**3**

شرح متغیر پیامد

فاکتور رشد شبه انسولینی

مقاطع زمانی اندازه‌گیری

قبل و بعد از اتمام مداخلات

نحوه اندازه‌گیری متغیر

با استفاده از کیت الایزا

**4**

شرح متغیر پیامد

هورمون رشد

مقاطع زمانی اندازه‌گیری

قبل و بعد از اتمام مداخلات

نحوه اندازه‌گیری متغیر

با استفاده از کیت الایزا

**5**

شرح متغیر پیامد

کورتیزول

مقاطع زمانی اندازه‌گیری

قبل و بعد از اتمام مداخلات

نحوه اندازه‌گیری متغیر

با استفاده از کیت الایزا

**6**

شرح متغیر پیامد

هموگلوبین گلیکوزیله

مقاطع زمانی اندازه‌گیری

قبل و بعد از اتمام مداخلات

نحوه اندازه‌گیری متغیر

با استفاده از کیت الایزا

**7**

شرح متغیر پیامد

قند حون ناشتا

مقاطع زمانی اندازه‌گیری

قبل و بعد از اتمام مداخلات

نحوه اندازه‌گیری متغیر

با استفاده از کیت الایزا

**8**

شرح متغیر پیامد

شمارش کامل خون

مقاطع زمانی اندازه‌گیری

قبل و بعد از اتمام مداخلات

نحوه اندازه‌گیری متغیر

با استفاده از کیت الایزا

**متغیر پیامد ثانویه**

**1**

شرح متغیر پیامد

کیفیت زندگی

مقاطع زمانی اندازه‌گیری

قبل و بعد از اتمام مداخلات

نحوه اندازه‌گیری متغیر

با استفاده از پرسشنامه

**2**

شرح متغیر پیامد

ترکیب بدن

مقاطع زمانی اندازه‌گیری

قبل و بعد از اتمام مداخلات

نحوه اندازه‌گیری متغیر

با استفاده از وزن، شاخص توده بدن، نسبت دور کمر به دور باسن و درصد چربی

**3**

شرح متغیر پیامد

آمادگی جسمانی

مقاطع زمانی اندازه‌گیری

قبل و بعد از اتمام مداخلات

نحوه اندازه‌گیری متغیر

با استفاده از اندازه گیری توان هوازی ، توان بی هوازی، تعادل، انعطاف و قدرت دست

**4**

شرح متغیر پیامد

فشار خون

مقاطع زمانی اندازه‌گیری

قبل و بعد از اتمام مداخلات

نحوه اندازه‌گیری متغیر

با استفاده از دستگاه فشار خون

**گروه‌های مداخله**

**1**

شرح مداخله

گروه مداخله: گروه تمرینات ترکیبی: شانزده هفته ، سه جلسه در هفته. تمرینات هوازی با شدت 50 تا 75 درصد حداکثر ضربان قلب، بیست دقیقه، تمرینات پیلاتس سه ست با 6 تا 12 تکرار و تمرینات با تحمل وزن بدن به مدت بیست دقیقه انجام شد.

طبقه بندی

شیوه زندگی

**2**

شرح مداخله

گروه شاهد: بدون مداخله

طبقه بندی

شیوه زندگی

**مراکز بیمار گیری**

**1**

مرکز بیمار گیری

**نام مرکز بیمار گیری**

بیمارستان کودکان هفده شهریور

**نام کامل فرد مسوول**

دکتر ستیلا دلیلی

**آدرس خیابان**

رشت - خیابان نامجو - خیابان شهید سیادتی

**شهر**

رشت

**استان**

گیلان

**کد پستی**

4147654919

**تلفن**

+98 13 3336 9070

**ایمیل**

marzieh.nazari.v@gmail.com

**حمایت کنندگان / منابع مالی**

**1**

حمایت کننده مالی

**نام سازمان / نهاد**

دانشگاه آزاد اسلامی

**نام کامل فرد مسوول**

علی دلپسند

**آدرس خیابان**

رشت - دروازه لاکان - دانشگاه آزاد اسلامی واحد رشت

**شهر**

رشت

**استان**

گیلان

**کد پستی**

۴۱۴۷۶۵۴۹۱۹

**تلفن**

+98 13 3342 2153

**ایمیل**

shabani_msn@yahoo.com

ردیف بودجه

کد بودجه

آیا منبع مالی همان سازمان یا نهاد حمایت کننده مالی است؟

بلی

عنوان منبع مالی

دانشگاه آزا د اسلامی

درصد تامین مالی مطالعه توسط این منبع

100

بخش عمومی یا خصوصی

خصوصی

مبدا اعتبار از داخل یا خارج کشور

داخلی

طبقه بندی منابع اعتبار خارحی

*خالی*

کشور مبدا

طبقه بندی موسسه تامین کننده اعتبار

دانشگاهی

**فرد مسوول پاسخگویی عمومی کارآزمایی**

اطلاعات تماس

**نام سازمان / نهاد**

دانشگاه آزاد اسلامی

**نام کامل فرد مسوول**

مرضیه نظری

**موقعیت شغلی**

دانشجوی دکتری

**آخرین مدرک تحصیلی**

فوق لیسانس

**سایر حوزه‌های کاری/تخصص‌ها**

فیزیولوژی ورزش

**آدرس خیابان**

رشت، پل تالشان، دانشگاه آزاد اسلامی واحد رشت

**شهر**

رشت

**استان**

گیلان

**کد پستی**

4147654919

**تلفن**

+98 13 3342 2153

**ایمیل**

marzieh.nazari.v@gmail.com

**فرد مسوول پاسخگویی علمی مطالعه**

اطلاعات تماس

**نام سازمان / نهاد**

دانشگاه آزاد اسلامی

**نام کامل فرد مسوول**

دکتر رامین شعبانی

**موقعیت شغلی**

دانشیار

**آخرین مدرک تحصیلی**

Ph.D.

**سایر حوزه‌های کاری/تخصص‌ها**

فیزیولوژی ورزش

**آدرس خیابان**

رشت، پل تالشان، دانشگاه آزاد اسلامی واحد رشت

**شهر**

رشت

**استان**

گیلان

**کد پستی**

4147654919

**تلفن**

+98 13 3342 2153

**ایمیل**

shabani_msn@yahoo.com

**فرد مسوول به‌روز رسانی اطلاعات**

اطلاعات تماس

**نام سازمان / نهاد**

دانشگاه آزاد اسلامی

**نام کامل فرد مسوول**

مرضیه نظری

**موقعیت شغلی**

دانشجوی دکتری

**آخرین مدرک تحصیلی**

فوق لیسانس

**سایر حوزه‌های کاری/تخصص‌ها**

فیزیولوژی ورزش

**آدرس خیابان**

رشت، پل تالشان، دانشگاه آزاد اسلامی واحد رشت

**شهر**

رشت

**استان**

گیلان

**کد پستی**

4147654919

**تلفن**

+98 13 3342 2153

**ایمیل**

marzieh.nazari.v@gmail.com

**برنامه انتشار**

فایل داده شرکت کنندگان (IPD)

هنوز تصمیم نگرفته‌ام - برنامه انتشار آن هنوز مشخص نیست

پروتکل مطالعه

هنوز تصمیم نگرفته‌ام - برنامه انتشار آن هنوز مشخص نیست

نقشه آنالیز آماری

مصداق ندارد

فرم رضایتنامه آگاهانه

هنوز تصمیم نگرفته‌ام - برنامه انتشار آن هنوز مشخص نیست

گزارش مطالعه بالینی

هنوز تصمیم نگرفته‌ام - برنامه انتشار آن هنوز مشخص نیست

کد‌های استفاده شده در آنالیز

مصداق ندارد

نظام دسته‌بندی داده (دیکشنری داده)

مصداق ندارد
