# Supplementary material for: Effect of concurrent resistance-aerobic training on inflammatory factors and growth hormones in children with type 1 diabetes: a randomized controlled clinical trial
Source: Trials. 2023 Aug 12;24:519. doi: 10.1186/s13063-023-07553-0 (PMC10422817; doi:10.1186/s13063-023-07553-0)
Supplement: Supplementary file 2 — Additional file 2. English protocol summary: English protocol. Ethics approval in Persian: Ethics approval in Persian. Ethics approval in English: Ethics approval in English. [file 13063_2023_7553_MOESM2_ESM.docx]

**Effect of combined exercise training on inflammatory factors and levels of hormones associated with blood glucose homeostasis in pediatric type 1 diabetes**

**Protocol summary**

Study aim

Effect of combined exercise training on inflammatory factors and levels of hormones associated with blood glucose homeostasis in pediatric type 1 diabetes

Design

In this research, 44 children with type 1 diabetes referring to 17 Shahrivar pediatric hospital were randomly assigned in the two groups. They will be divided into two groups; 22 people in experimental and 22 people in control group.

Settings and conduct

The present study is a semi-experimental study among the child with type 1 diabetes of city of Rasht for 16 weeks. Blood sampling is performed after 12 hours of fasting and before and after 16 weeks of concurrent training.

Participants/Inclusion and exclusion criteria

Inclusion criteria: Having age ranges of 8-15 years, A1c≥7, Pediatric type 1 diabetes, No regular exercise Exclusion criteria: more than three session absence from the training

Intervention groups

Intervention group: Combined training group. Aerobic training: sixteen weeks, three times per week. Aerobic exercises were performed with an intensity of 50-75% of maximum heart rate, pilates exercises, 3 set, 6-12 repeat and training using body weight with 20 minutes. Control group: Without intervention

Main outcome variables

IL-1B, HS-CRP, IGF-1, Grows hormone, Cortisol, HbA1c, FBS, CBC

**General information**

Reason for update

Acronym

IRCT registration information

IRCT registration number: **IRCT20150531022498N30**

Registration date: **2019-07-26, 1398/05/04**

Registration timing: **retrospective**

Last update: **2019-07-26, 1398/05/04**

Update count: **0**

Registration date

2019-07-26, 1398/05/04

Registrant information

**Name**

Ramin Shabani

**Name of organization / entity**

Islamic Azad University

**Country**

Iran (Islamic Republic of)

**Phone**

+98 13 3375 2715

**Email address**

shabani@iaurasht.ac.ir

Recruitment status

**Recruitment complete**

Funding source

Expected recruitment start date

2019-02-09, 1397/11/20

Expected recruitment end date

2019-07-22, 1398/04/31

Actual recruitment start date

empty

Actual recruitment end date

empty

Trial completion date

empty

Scientific title

Effect of combined exercise training on inflammatory factors and levels of hormones associated with blood glucose homeostasis in pediatric type 1 diabetes

Public title

Effect of combined exercise training in pediatric type 1 diabetes

Purpose

Prevention

Inclusion/Exclusion criteria

**Inclusion criteria:**

Having age ranges of 8-15 years A1c≥7 Pediatric type 1 diabetes No regular exercise

**Exclusion criteria:**

More than three session absence from the training

Age

From **8 years** old to **15 years** old

Gender

Both

Phase

N/A

Groups that have been masked

*No information*

Sample size

Target sample size: **44**

Randomization (investigator's opinion)

Randomized

Randomization description

Subjects were divided into 2 groups (one control group and one experimental groups) using the simple random method. This sampling will be done based on the red and blue balls inside the box that are randomly taken by the subjects (Red Orb and the Blue Orb of the experimental group).

Blinding (investigator's opinion)

Not blinded

Blinding description

Placebo

Not used

Assignment

Parallel

Other design features

**Secondary Ids**

empty

**Ethics committees**

**1**

Ethics committee

**Name of ethics committee**

Ethics Committee of Islamic Azad University Rasht Branch

**Street address**

Islamic Azad University Rasht Branch, Taleshan Bridge, Rasht

**City**

Rasht

**Province**

Guilan

**Postal code**

۴۱۴۷۶۵۴۹۱۹

Approval date

2019-07-19, 1398/04/28

Ethics committee reference number

IR.IAU.RASHT.REC.1398.011

**2**

Ethics committee

**Name of ethics committee**

Ethics Committee of Islamic Azad University Rasht Branch

**Street address**

Islamic Azad University Rasht Branch, Taleshan Bridge, Rasht

**City**

Rasht

**Province**

Guilan

**Postal code**

۴۱۴۷۶۵۴۹۱۹

Approval date

2017-09-19, 1396/06/28

Ethics committee reference number

IR.IAU.RASHT.REC.1396.168

**Health conditions studied**

**1**

Description of health condition studied

Type 1 diabetes

ICD-10 code

E10

ICD-10 code description

Type 1 diabetes mellitus

**Primary outcomes**

**1**

Description

IL-1B

Timepoint

Before and after the end of interventions

Method of measurement

using ELISA Kit

**2**

Description

High-sensitivity C-reactive Protein (hs-CRP)

Timepoint

Before and after the end of interventions

Method of measurement

using ELISA Kit

**3**

Description

IGF-1

Timepoint

Before and after the end of interventions

Method of measurement

using ELISA Kit

**4**

Description

Growth hormone

Timepoint

Before and after the end of interventions

Method of measurement

using ELISA Kit

**5**

Description

Cortisol

Timepoint

Before and after the end of interventions

Method of measurement

using ELISA Kit

**6**

Description

HbA1c

Timepoint

Before and after the end of interventions

Method of measurement

using ELISA Kit

**7**

Description

Fast blood sugar

Timepoint

Before and after the end of interventions

Method of measurement

using ELISA Kit

**8**

Description

Complete Blood Count (CBC)

Timepoint

Before and after the end of interventions

Method of measurement

using ELISA Kit

**Secondary outcomes**

**1**

Description

Quality of Life

Timepoint

Before and after the end of interventions

Method of measurement

Using questionnaire

**2**

Description

Body composition

Timepoint

Before and after the end of interventions

Method of measurement

Using weight, body mass index, waist to hip ratio and fat percent

**3**

Description

Physical fitness

Timepoint

Before and after the end of interventions

Method of measurement

Using measurement of aerobic power ,anaerobic , balance ,Flexibility and hand grip

**4**

Description

Blood pressure

Timepoint

Before and after the end of interventions

Method of measurement

Using the blood pressure device

**Intervention groups**

**1**

Description

Intervention group: Combined training group. Aerobic training: sixteen weeks, three times per week . Aerobic exercises were performed with an intensity of 50-75% of maximum heart rate ,pilates exercises, 3 set, 6-12 repeat and training using body weight with 20 minuets.

Category

Lifestyle

**2**

Description

Control group: Without intervention

Category

Lifestyle

**Recruitment centers**

**1**

Recruitment center

**Name of recruitment center**

17 Shahrivar Children's Hospital

**Full name of responsible person**

Dr setila dalili

**Street address**

Shahid Siadati Avenu, Namjoo Street, Rasht

**City**

Rasht

**Province**

Guilan

**Postal code**

4147654919

**Phone**

+98 13 3336 9070

**Email**

marzieh.nazari.v@gmail.com

**Sponsors / Funding sources**

**1**

Sponsor

**Name of organization / entity**

Islamic Azad University

**Full name of responsible person**

Ali delpasand

**Street address**

Islamic Azad University, Rasht Brunch

**City**

Rasht

**Province**

Guilan

**Postal code**

۴۱۴۷۶۵۴۹۱۹

**Phone**

+98 13 3342 2153

**Email**

shabani_msn@yahoo.com

Grant name

Grant code / Reference number

Is the source of funding the same sponsor organization/entity?

Yes

Title of funding source

Islamic Azad University

Proportion provided by this source

100

Public or private sector

Private

Domestic or foreign origin

Domestic

Category of foreign source of funding

*empty*

Country of origin

Type of organization providing the funding

Academic

**Person responsible for general inquiries**

Contact

**Name of organization / entity**

Islamic Azad University

**Full name of responsible person**

Marzieh Nazari

**Position**

PhD student

**Latest degree**

Master

**Other areas of specialty/work**

Exercise physiology

**Street address**

Islamic Azad University Rasht Branch, Taleshan Bridge, Rasht

**City**

Rasht

**Province**

Guilan

**Postal code**

4147654919

**Phone**

+98 13 3342 2153

**Email**

marzieh.nazari.v@gmail.com

**Person responsible for scientific inquiries**

Contact

**Name of organization / entity**

Islamic Azad University

**Full name of responsible person**

Dr Ramin shabani

**Position**

Associate Professor

**Latest degree**

Ph.D.

**Other areas of specialty/work**

Exercise physiology

**Street address**

Islamic Azad University Rasht Branch, Taleshan Bridge, Rasht

**City**

Rasht

**Province**

Guilan

**Postal code**

4147654919

**Phone**

+98 13 3342 2153

**Email**

shabani_msn@yahoo.com

**Person responsible for updating data**

Contact

**Name of organization / entity**

Islamic Azad University

**Full name of responsible person**

Marzieh Nazari

**Position**

PhD Student

**Latest degree**

Master

**Other areas of specialty/work**

Exercise physiology

**Street address**

Islamic Azad University Rasht Branch, Taleshan Bridge, Rasht

**City**

Rasht

**Province**

Guilan

**Postal code**

4147654919

**Phone**

+98 13 3342 2153

**Email**

marzieh.nazari.v@gmail.com

**Sharing plan**

Deidentified Individual Participant Data Set (IPD)

Undecided - It is not yet known if there will be a plan to make this available

Study Protocol

Undecided - It is not yet known if there will be a plan to make this available

Statistical Analysis Plan

Not applicable

Informed Consent Form

Undecided - It is not yet known if there will be a plan to make this available

Clinical Study Report

Undecided - It is not yet known if there will be a plan to make this available

Analytic Code

Not applicable

Data Dictionary

Not applicable
